# Impact of atherosclerosis imaging-quantitative computed tomography on diagnostic certainty, downstream testing, coronary revascularization, and medical therapy: the CERTAIN study

**DOI:** 10.1093/ehjci/jeae029

**Published:** 2024-01-25

**Authors:** Nick S Nurmohamed, Jason H Cole, Matthew J Budoff, Ronald P Karlsberg, Himanshu Gupta, Lance E Sullenberger, Carlos G Quesada, Habib Rahban, Kevin M Woods, Jeffrey R Uzzilia, Scott L Purga, Melissa Aquino, Udo Hoffmann, James K Min, James P Earls, Andrew D Choi

**Affiliations:** Department of Cardiology, Amsterdam UMC, Vrije Universiteit Amsterdam, Meibergdreef 9, 1105 AZ Amsterdam, The Netherlands; Department of Vascular Medicine, Amsterdam UMC, University of Amsterdam, Meibergdreef 9, 1105 AZ Amsterdam, The Netherlands; Division of Cardiology, Department of Radiology, The George Washington University School of Medicine, Washington, DC, USA; Cardiology Associates of Mobile, Mobile, AL, USA; Lundquist Institute at Harbor-UCLA Medical Center, Torrance, CA, USA; Cardiovascular Research Foundation of Southern California, Cedars-Sinai Heart Institute, Beverly Hills, CA; Division of Cardiac Imaging, Valley Heart and Vascular Institute, Valley Health System, Ridgewood, NJ, USA; Capital Cardiology Associates, Albany, NY, USA; Cardiovascular Research Foundation of Southern California, Cedars-Sinai Heart Institute, Beverly Hills, CA; Cardiovascular Research Foundation of Southern California, Cedars-Sinai Heart Institute, Beverly Hills, CA; Capital Cardiology Associates, Albany, NY, USA; Capital Cardiology Associates, Albany, NY, USA; Capital Cardiology Associates, Albany, NY, USA; Cleerly Inc., Denver, CO, USA; Cleerly Inc., Denver, CO, USA; Cleerly Inc., Denver, CO, USA; Division of Cardiology, Department of Radiology, The George Washington University School of Medicine, Washington, DC, USA; Cleerly Inc., Denver, CO, USA; Division of Cardiology, Department of Radiology, The George Washington University School of Medicine, Washington, DC, USA

**Keywords:** coronary CT angiography, CCTA, atherosclerosis imaging-quantitative computed tomography, AI-QCT, artificial intelligence, multi-centre

## Abstract

**Aims:**

The incremental impact of atherosclerosis imaging-quantitative computed tomography (AI-QCT) on diagnostic certainty and downstream patient management is not yet known. The aim of this study was to compare the clinical utility of the routine implementation of AI-QCT versus conventional visual coronary CT angiography (CCTA) interpretation.

**Methods and results:**

In this multi-centre cross-over study in 5 expert CCTA sites, 750 consecutive adult patients referred for CCTA were prospectively recruited. Blinded to the AI-QCT analysis, site physicians established patient diagnoses and plans for downstream non-invasive testing, coronary intervention, and medication management based on the conventional site assessment. Next, physicians were asked to repeat their assessments based upon AI-QCT results. The included patients had an age of 63.8 ± 12.2 years; 433 (57.7%) were male. Compared with the conventional site CCTA evaluation, AI-QCT analysis improved physician’s confidence two- to five-fold at every step of the care pathway and was associated with change in diagnosis or management in the majority of patients (428; 57.1%; *P* < 0.001), including for measures such as Coronary Artery Disease-Reporting and Data System (CAD-RADS) (295; 39.3%; *P* < 0.001) and plaque burden (197; 26.3%; *P* < 0.001). After AI-QCT including ischaemia assessment, the need for downstream non-invasive and invasive testing was reduced by 37.1% (*P* < 0.001), compared with the conventional site CCTA evaluation. Incremental to the site CCTA evaluation alone, AI-QCT resulted in statin initiation/increase an aspirin initiation in an additional 28.1% (*P* < 0.001) and 23.0% (*P* < 0.001) of patients, respectively.

**Conclusion:**

The use of AI-QCT improves diagnostic certainty and may result in reduced downstream need for non-invasive testing and increased rates of preventive medical therapy.

## Graphical Abstract

**Graphical Abstract:**
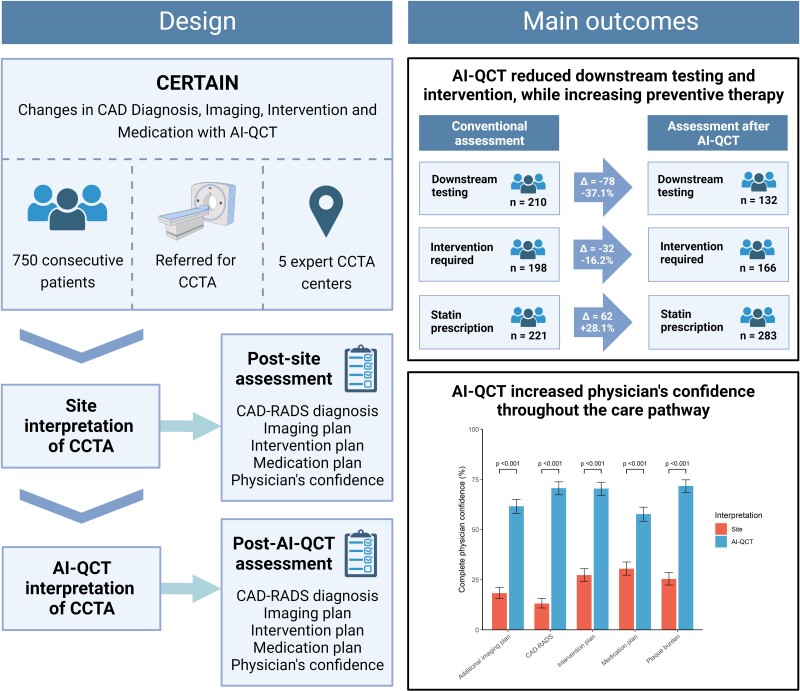
Design and main outcomes of the CERTAIN study. In this multi-centre study, the use of AI-QCT improved diagnostic certainty, reduced the downstream need for non-invasive testing and coronary intervention, and increased rates of preventive medical therapy. AI-QCT, atherosclerosis imaging-quantitative computed tomography; CAD, coronary artery disease; CAD-RADS, Coronary Artery Disease-Reporting and Data System; CCTA, coronary CT angiography; CERTAIN, Changes in CAD Diagnosis, Imaging, Intervention and Medication with AI-QCT. Created with BioRender.com.

## Introduction

In the recent international guidelines on the evaluation of patients suspected of coronary artery disease (CAD),^1,2^ coronary CT angiography (CCTA) assumes a pivotal role in identifying patients with obstructive CAD as well as the estimation of risk for future cardiovascular events. The recently updated Coronary Artery Disease-Reporting and Data System (CAD-RADS) 2.0^3^ has taken the first step of including a measure of atherosclerotic plaque burden in addition to stenosis presence, given the extensive evidence for its prognostic and therapeutic relevance.^4,5^ However, at present, manual grading of coronary stenosis and atherosclerosis is labour-intensive and associated with a high inter-observer variability between expert clinical readers as well as overestimation of coronary stenosis.^4,6^

Given the complexity of coronary atherosclerosis assessment and training required, it is questionable whether routine human analysis can adequately weigh all previously reported relevant CCTA parameters, such as high-risk plaque characteristics,^7,8^ low-attenuation plaque volumes,^5^ and other plaque measures; and whether additional technology tools may be required. Atherosclerosis imaging-quantitative computed tomography (AI-QCT) is an artificial intelligence-enabled approach that performs whole-heart evaluation of all coronary vessels for atherosclerosis, stenosis, and ischaemia. AI-QCT has demonstrated robust prognostic utility for near-, intermediate-, and long-term cardiovascular events.^4,9–12^ Moreover, AI-QCT_ISCHEMIA_ has shown accurate performance for diagnosis of coronary ischaemia in a preliminary analysis.^13^

To date, the clinical utility of implementing AI-QCT beyond conventional expert CCTA evaluation has not been evaluated. The aim of this study was to investigate the clinical utility of routine implementation of AI-QCT in comparison to conventional visual assessment of CCTA by expert readers.

## Methods

### Study design and population

The Changes in CAD Diagnosis, Imaging, Intervention and Medication with AI-QCT (CERTAIN) was a multi-centre study in five expert CCTA centres. All outpatient adults who received a clinically indicated CCTA for cardiac symptoms were consecutively included. After undergoing CCTA, the exam was first analysed according to site protocols by a Level III cardiologist and/or radiologist. Treating physicians were asked to complete an assessment of the patients’ diagnosis, additional downstream non-invasive imaging plan, coronary intervention plan, and medication management based on the conventional site assessment, blinded to the AI-QCT results. Subsequently, CCTA exams were analysed using AI-QCT, after which physicians were asked to repeat the assessment. A total of 775 patients were included, of whom 25 were excluded because the CT scans could not be uploaded for AI-QCT analysis, resulting in a final study population of 750 patients with AI-QCT evaluation.

### Questionnaires following site and AI-QCT assessment

Following both conventional CCTA interpretation and AI-QCT performance, physicians responded to questionnaires that addressed: CAD-RADS, plaque status, downstream testing plan, interventional plan, and medication prescription. When responding to questions related to conventional CCTA interpretation, physicians were blinded to AI-QCT results. After completing questionnaires on conventional CCTA interpretation, physicians received access to the AI-QCT results. In both cases, physicians had full access to the patient’s medical history, laboratory data, and other prior diagnostic tests when responding to study questionnaires. For each category in the questionnaire, physicians were also asked to grade their level of confidence using a quantitative Likert scale ranging from 1–5 (1, not confident at all; 2, slightly confident; 3, somewhat confident; 4, fairly confident; 5, completely confident). Prior to the study initiation, physicians at the participating sites had experience with the use of AI-QCT and were trained in the use of the AI-QCT_ISCHEMIA_ algorithm.

### CCTA acquisition and site assessment

All patients underwent CCTA imaging with multi-detector row CT scanners of ≥64 rows, preferably using a prospectively ECG-gated CCTA protocol. CCTA acquisition was performed in accordance with the Society of Cardiovascular Computed Tomography (SCCT) guidelines.^14^ Site assessment was performed by board-certified Level III cardiologists or radiologists according to the standard clinical practice and following the most recent guidelines for the Society of Cardiovascular Computed Tomography CCTA interpretation.^3^

### AI-QCT analysis

An FDA-cleared, artificial intelligence-based software approach was used to analyse the CCTA images (AI-QCT; Cleerly Inc., Denver, CO, USA).^9^ This FDA-cleared software service utilizes a series of validated convolutional neural networks for image quality assessment, coronary segmentation and labelling, lumen wall evaluation, vessel contour determination, and plaque characterization. Prior validation of AI-QCT has been reported in multi-centre trials using expert consensus, quantitative coronary angiography, and fractional flow reserve (FFR) as previously published,^9,10,12^ as well as intravascular ultrasound.^15^ The algorithm first produces a centreline, lumen, and outer vessel wall contouring for every phase available and subsequently selects the optimal series for analysis. The choice for best quality image is then made on a per-vessel basis. After automated segmentation and labelling in all vessels, plaques are characterized and quantified based on the Hounsfield unit (HU) attenuation. Finally, a trained radiologic technologist provides a quality assurance overview of the AI-QCT analysis, and can make adjustments if necessary.

Coronary segments with a diameter ≥1.5 mm were included in the analysis using the modified 18-segment SCCT model.^16^ Coronary percentage stenosis was adjudicated on a per-vessel basis as per SCCT guidelines and categorized by the Coronary Artery Disease-Reporting and Data System (CAD-RADS).^3^ Each segment was evaluated for the presence or absence of coronary atherosclerosis, defined as any tissue structure >1 mm^2^ within the coronary artery wall that was differentiated from the surrounding epicardial tissue, epicardial fat, or the vessel lumen itself. Plaque volumes (mm^3^) were calculated for each coronary lesion and then summed to compute the total plaque volume at the segment, vessel, and patient level. Plaque volume was categorized using Hounsfield unit (HU) ranges, with low-density non-calcified plaque (LD-NCP) defined as plaques with any component on a pixel-level basis and quantified on an increment of 0.1 mm^3^ as <30 HU, non-calcified plaque volume (NCPV) defined as HU between −30 and +350, and calcified plaque volume (CPV) defined as >350 HU.^17^ The coronary plaque volume was normalized to the total per-patient vessel volume to account for variation in the coronary artery volume, calculated as the plaque volume/vessel volume × 100%. These normalized volumes were reported as the per cent atheroma volume (PAV), per cent NCPV, and per cent CPV. Plaque volumes were categorized according to a previously reported plaque staging system, into Stage 0 (Normal, 0 ​mm^3^ TPV), Stage 1 (Mild, > 0–250 ​mm^3^ TPV), Stage 2 (Moderate, > 250–750 ​mm^3^ TPV), and Stage 3 (Severe, > 750 mm^3^ TPV).^4,18^

AI-QCT also diagnoses coronary vessel-specific ischaemia (AI-QCT_ISCHEMIA_). The AI-QCT_ISCHEMIA_ algorithm is a random forest model integrating 37 atherosclerotic and vascular morphologic parameters to predict the presence of reduced FFR using a binary threshold (presence or absence of coronary ischaemia).^13^ The machine-learned model has been validated in multi-centre validation cohorts vs. invasive FFR.^13^

### Statistical analysis

Data are presented as mean ± standard deviation for normally distributed variables or median with interquartile range (IQR) for non-normally distributed data. The normality of data distribution was assessed using histograms and probability plots. Categorical variables are expressed as absolute numbers and percentages. The Wilcoxon signed-rank test was used to test the differences in proportions in CAD-RADS assessment, plaque burden assessment, downstream testing plan, and interventional plan between the conventional CCTA and the AI-QCT assessment. To compare the percentages of complete physicians’ confidence between the two groups, McNemar tests were used. All statistical analyses were performed using RStudio software version 4.0.3 (R Foundation, Vienna, Austria) and SAS software for Windows version 9.4 (SAS Institute Inc., Cary, NC, USA).

## Results

### Baseline characteristics

**Figure 1:**
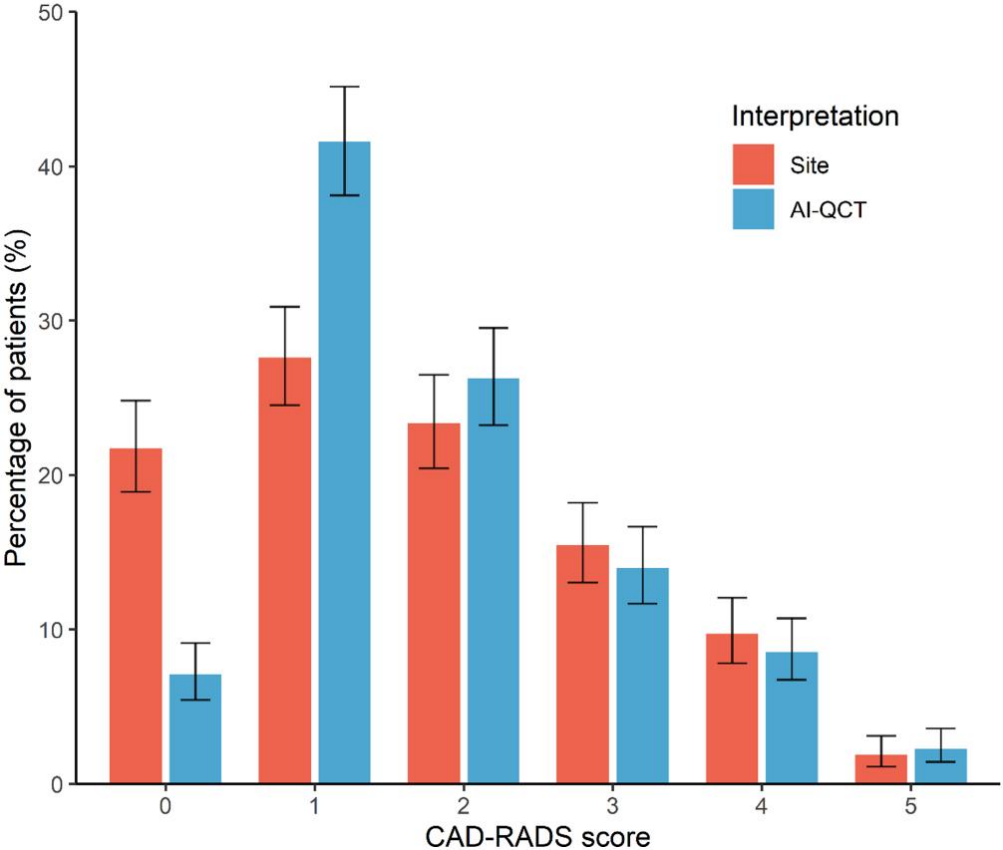
Comparison of CAD-RADS assessment between site readers and after AI-QCT employment. CAD-RADS score categories after conventional site interpretation and after AI-QCT interpretation. Shown are percentages with 95% confidence interval. AI-QCT, atherosclerosis imaging-quantitative computed tomography; CAD-RADS, Coronary Artery Disease-Reporting and Data System.

The 750 consecutive study patients had a mean age of 63.8 ± 12.2 years, and 433 (57.7%) were male (*Table 1*). The study patients were predominantly White (575; 76.7%), while 44 (5.9%) patients were Asian and 42 (5.6%) were Black. CAD risk factors were highly prevalent: patients had a systolic blood pressure of 136 ± 48 mmHg, 136 (18.3%) patients had Type 2 diabetes mellitus, 282 (37.8%) had a history of smoking, and 366 (49.1%) had a family history of CAD. Prior to CCTA imaging, nearly half of patients were on statin therapy (364; 48.5%), 294 (39.2%) patients were using aspirin, while 190 (25.3%) patients were on beta-blocker therapy. According to the conventional site CCTA interpretation, 203 (27.1%) patients had obstructive stenosis, compared with 181 (24.0%) patients diagnosed with obstructive stenosis according to the AI-QCT interpretation (*Figure 1*). According to the AI-QCT coronary artery plaque staging system, 5 (0.7%) were categorized as Stage 0 (0 mm^3^), 452 (60.3%) as Stage 1 (>0–250 mm^3^), 211 (28.1%) as Stage 2 (>250–750 mm^3^), and 82 (10.9%) as Stage 3 (>750 mm^3^).

**Table 1:** Baseline characteristics

Characteristic	Overall
	*n* = 750
Age at baseline, years	63.8 ± 12.2
Male sex	433 (57.7%)
Race	
White	575 (76.7%)
Asian	44 (5.9%)
Black	42 (5.6%)
Other/unknown	89 (11.9%)
BMI, kg/m^2^	28.5 ± 5.5
Systolic blood pressure, mmHg	136 ± 48
Diastolic blood pressure, mmHg	81 ± 12
Type 2 diabetes mellitus	136 (18.3%)
Smoking history	282 (37.8%)
Family history of CAD	366 (49.1%)
Use of medication prior to CCTA	
Aspirin	294 (39.2%)
Beta-blocker	190 (25.3%)
ACE inhibitor	104 (13.9%)
Long-acting nitrates	164 (21.9%)
Statin	364 (48.5%)
Low–moderate intensity	234 (31.2%)
High intensity	130 (17.3%)
Ezetimibe	50 (6.7%)
PCSK9 inhibitor	41 (5.5%)
Icosapent ethyl	42 (5.6%)
CAD-RADS score (based on AI-QCT)	
CAD-RADS 0	9 (1.2%)
CAD-RADS 1	339 (45.2%)
CAD-RADS 2	221 (29.5%)
CAD-RADS 3	124 (16.5%)
CAD-RADS 4	39 (5.2%)
CAD-RADS 5	18 (2.4%)
Total plaque volume, mm^3^	160.4 (53.9–426.4)
Non-calcified plaque volume, mm^3^	104.3 (41.6–222.8)
Calcified plaque volume, mm^3^	39.2 (1.3–179.4)
Per cent atheroma volume, %	4.9 (1.7–13.0)
Per cent non-calcified plaque volume	3.3 (1.4–6.5)
Per cent calcified plaque volume	1.2 (0.0–5.7)
Plaque stage	
Stage 0 (0 mm^3^)	5 (0.7%)
Stage 1 (>0–250 mm^3^)	452 (60.3%)
Stage 2 (>250–750 mm^3^)	211 (28.1%)
Stage 3 (>750 mm^3^)	82 (10.9%)
Presence of two feature positive plaque	209 (27.9%)
AI-QCT_ISCHEMIA_ positive	162 (21.6%)
Image quality score (Likert scale)	
1—poor	0 (0.0%)
2—fair	52 (6.9%)
3—good	378 (50.4%)
4—very good	299 (39.9%)
5—excellent	21 (2.8%)
Mean ± SD; median (IQR); *n* (%)	

### Change in physician interpretation of CAD-RADS and plaque burden

**Figure 2:**
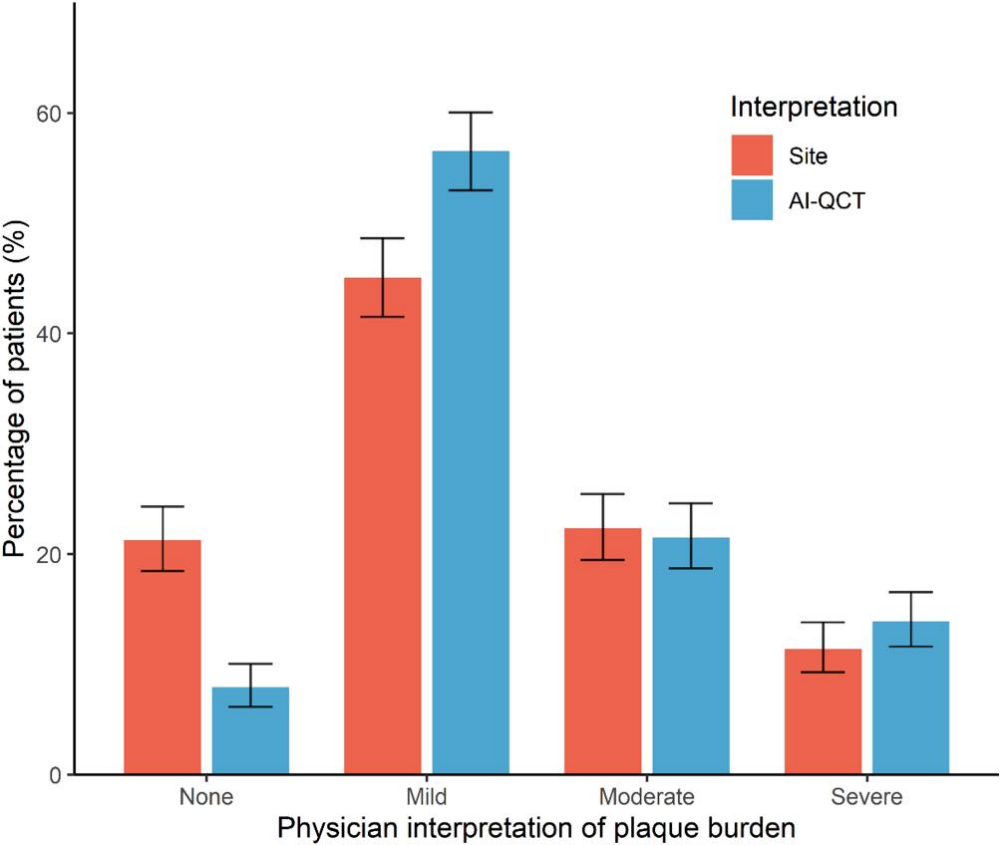
Comparison physician’s plaque burden assessment between site readers and after AI-QCT employment. Interpretation of plaque burden according to treating physicians after conventional site interpretation and after AI-QCT interpretation. Shown are percentages with 95% confidence interval. AI-QCT, atherosclerosis imaging-quantitative computed tomography; CAD-RADS, Coronary Artery Disease-Reporting and Data System.

Compared with the conventional site CCTA interpretation, evaluation with AI-QCT resulted in physicians modifying their CAD-RADS score diagnosis in 295 (39.3%) patients (*Tables 2* and *3*; *Figure 1*; *P* < 0.001). The qualitative physician assessment of overall plaque burden was also impacted by AI-QCT, with plaque burden changing in 197 (26.3%) patients (*Tables 2* and *3*; *Figure 2*; *P* < 0.001). With AI-QCT, the number of patients with no plaque according to the physician decreased from 159 (21.2%) to 58 (7.8%) patients (*P* < 0.001), while the number of patients with mild plaque increased with 27.7% (*P* < 0.001) from 336 (45.0%) to 429 (57.4%). The number of patients with moderate and severe plaque did not differ between the site and AI-QCT interpretation.

**Table 2:** Reclassification tables of AI-QCT assessment compared with conventional site assessment for CAD-RADS and plaque burden

CAD-RADS interpretation								
	AI-QCT							
CCTA	Indet.	0	1	2	3	4	5	Total
Indet.	2 (0.3%)	0 (0.0%)	0 (0.0%)	0 (0.0%)	0 (0.0%)	0 (0.0%)	0 (0.0%)	2 (0.3%)
0	0 (0.0%)	53 (7.1%)	107 (14.3%)	3 (0.4%)	0 (0.0%)	0 (0.0%)	0 (0.0%)	163 (21.7%)
1	0 (0.0%)	0 (0.0%)	176 (23.5%)	30 (4.0%)	1 (0.1%)	0 (0.0%)	0 (0.0%)	207 (27.6%)
2	0 (0.0%)	0 (0.0%)	27 (3.6%)	112 (14.9%)	30 (4.0%)	6 (0.8%)	0 (0.0%)	175 (23.3%)
3	0 (0.0%)	0 (0.0%)	2 (0.3%)	46 (6.1%)	54 (7.2%)	12 (1.6%)	2 (0.3%)	116 (15.5%)
4	0 (0.0%)	0 (0.0%)	0 (0.0%)	6 (0.8%)	20 (2.7%)	45 (6.0%)	2 (0.3%)	73 (9.7%)
5	0 (0.0%)	0 (0.0%)	0 (0.0%)	0 (0.0%)	0 (0.0%)	1 (0.1%)	13 (1.7%)	14 (1.9%)
Total	2 (0.3%)	53 (7.1%)	312 (41.6%)	197 (26.3%)	105 (14.0%)	64 (8.5%)	17 (2.3%)	750 (100%)

Plaque interpretation						
	AI-QCT					
CCTA	Indet.	None	Mild	Moderate	Severe	Total
Indet.	3 (0.4%)	0 (0.0%)	0 (0.0%)	0 (0.0%)	0 (0.0%)	3 (0.4%)
None	0 (0.0%)	55 (7.3%)	104 (13.9%)	0 (0.0%)	0 (0.0%)	159 (21.2%)
Mild	0 (0.0%)	3 (0.4%)	297 (39.6%)	31 (4.1%)	5 (0.7%)	336 (44.8%)
Moderate	0 (0.0%)	0 (0.0%)	25 (3.3%)	132 (17.6%)	10 (1.3%)	167 (22.3%)
Severe	0 (0.0%)	0 (0.0%)	3 (0.4%)	6 (0.8%)	76 (10.1%)	85 (11.3%)
Total	3 (0.4%)	58 (7.7%)	429 (57.2%)	169 (22.5%)	91 (12.1%)	750 (100%)

AI-QCT, atherosclerosis imaging-quantitative computed tomography; CAD-RADS, Coronary Artery Disease-Reporting and Data System; CCTA, coronary computed tomography angiography, Indet., indetermined.

**Table 3:** Change throughout the care pathway with AI-QCT compared with conventional site assessment

Care pathway component	Overall *n* = 750	Non-obstructive CAD (CAD-RADS 0–2)	Obstructive CAD (CAD-RADS 3–5)	*P*-value
		*n* = 545	*n* = 203	
Change in CAD-RADS score	295 (39.3%)	204 (37.0%)	91 (44.8%)	0.066
Change in plaque burden interpretation	197 (26.3%)	154 (28.3%)	43 (21.2%)	0.051
Change in imaging plan	175 (23.3%)	41 (7.5%)	133 (65.5%)	<0.001
Change in intervention plan	127 (16.9%)	31 (5.7%)	95 (46.8%)	<0.001
Change in medication prescription	173 (23.1%)	140 (25.7%)	33 (16.3%)	0.007
Overall net change	428 (57.1%)	268 (49.2%)	159 (78.3%)	<0.001

Two patients had an indeterminate CAD-RADS category and were omitted from the two categories. Patients were divided into non-obstructive and obstructive CAD based on the site CCTA interpretation. AI-QCT, atherosclerosis imaging-quantitative computed tomography; CAD-RADS, Coronary Artery Disease-Reporting and Data System.

### Change in downstream testing and intervention plan

**Figure 3:**
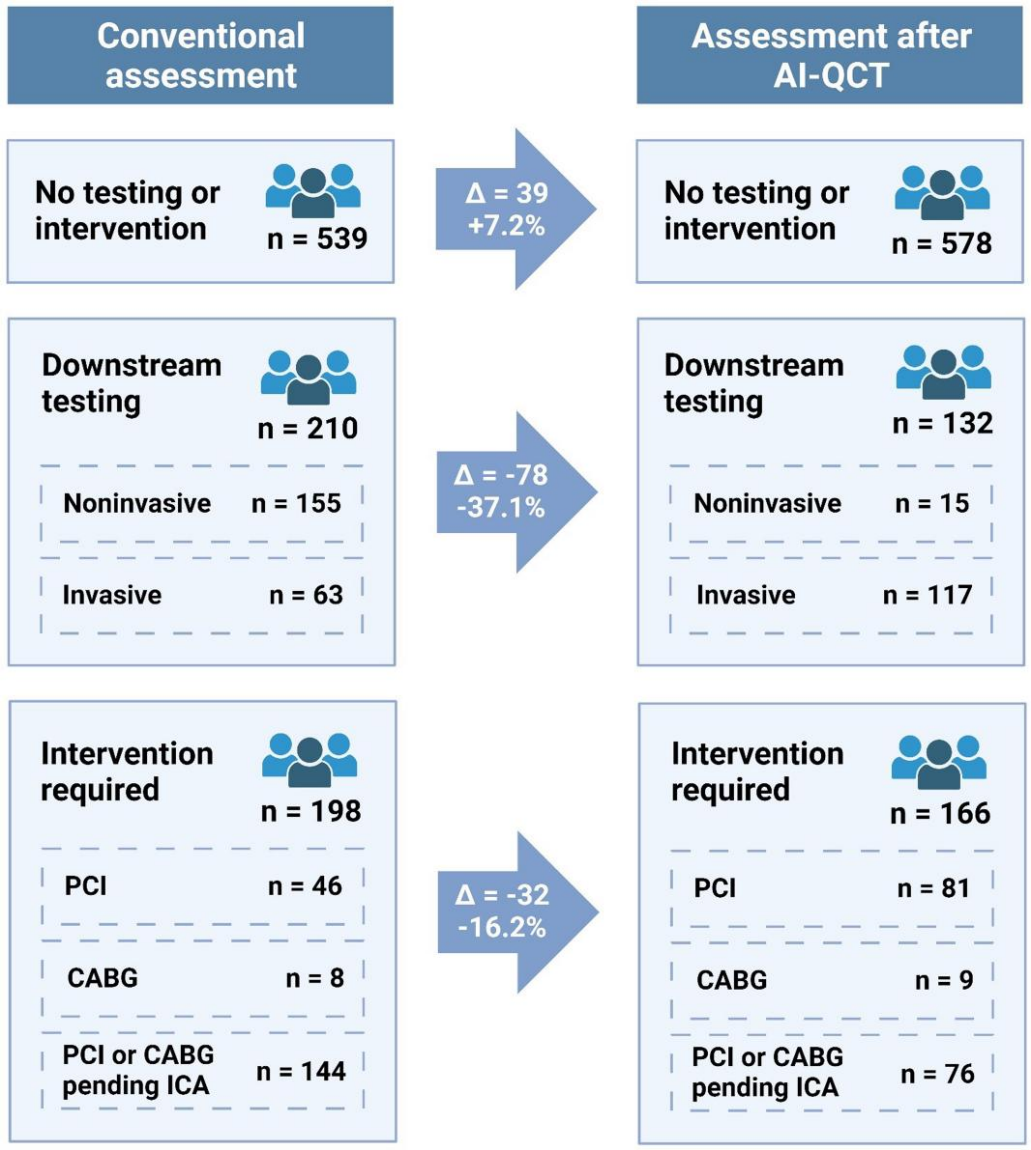
Change in downstream testing and intervention plan upon assessment with AI-QCT. Impact of AI-QCT application on downstream testing. For downstream testing, physicians had the possibility to choose both non-invasive and invasive testing. AI-QCT, atherosclerosis imaging-quantitative computed tomography; ECG, electrocardiogram; FFR_CT_, fractional flow reserve from CT; ICA, invasive coronary angiography; PET, positron emission tomography; SPECT, single photon emission computed tomography; CABG, coronary artery bypass grafting; PCI, percutaneous coronary intervention. Created with BioRender.com.

After AI-QCT assessment, physicians significantly altered their plan for downstream testing and intervention (*Figure 3*). With AI-QCT assessment, there was a change in imaging plan and intervention plan in 175 (23.3%) and 127 (16.9%) patients, respectively, compared with the conventional site assessment. Upon the site assessment of the CCTA, 155 (20.7%) patients were planned for downstream testing, of whom 44 (5.8%) were planned to undergo stress testing, while 141 (18.8%) were planned for FFR_CT_ measurement. After the AI-QCT assessment, the overall need for downstream testing was reduced with 37.1% (*P* < 0.001; *Figure 3*). Additionally, the intervention plan after the site CCTA interpretation was compared with the plan after AI-QCT analysis including ischaemia. Based on the site CCTA interpretation, 198 (26.4%) patients were recommended to undergo intervention, which consisted of percutaneous coronary intervention (PCI) in 46 (6.1%) patients, coronary artery bypass grafting (CABG) in 8 (1.1%) patients; for 144 (19.2%) patients, the type of intervention was indeterminate after CCTA interpretation. In contrast, after AI-QCT, the overall number of patients requiring intervention decreased by 16.2% (*P* < 0.001), with the type of intervention planned demonstrating higher rates of determination: 81 (10.8%) patients were planned to undergo PCI, 9 (1.2%) patients were planned for CABG, and in 66 (8.8%) patients, the type of intervention was still indeterminate pending invasive coronary angiography (ICA). According to the physician’s CCTA interpretation, 539 (71.9%) patients did not need further downstream testing or intervention, which increased with 7.2% (*n* = 39) to 578 (77.1%) with AI-QCT (*P* < 0.001).

### Change in prescription of preventive medication

CCTA findings by both conventional CCTA evaluation and AI-QCT resulted in planned changes for preventive medical treatment in a high number of patients (*Table 4*). For statin therapy, after the initial CCTA site assessment, 221 (29.5%) patients were started on a statin or were prescribed a dose increase. Following the AI-QCT interpretation, the number of patients prescribed a statin (dose increment) increased by 28.1% to 283 patients. A total of 235 (31.5%) patients were prescribed aspirin therapy after site assessment, which increased by 23.0% to 289 patients after AI-QCT assessment. The prescription of beta-blockers and ACE inhibitors also increased with 34.8 and 9.1%, respectively (*Table 4*). Overall, there was a change in the medication prescription in 173 (23.1%) patients with AI-QCT assessment compared with the conventional site assessment (*P* < 0.001).

**Table 4:** Increase in medication prescription after AI-QCT assessment

	Site	AI-QCT	Change (%)
Start or increase statin dose	221	283	62 (28.1%)
Start aspirin	235	289	54 (23.0%)
Start or increase beta-blocker	23	31	8 (34.8%)
Start or increase ACE inhibitor	22	24	2 (9.1%)

Shown is the number of patients. AI-QCT, atherosclerosis imaging-quantitative computed tomography; ACE, angiotensin-converting enzyme.

### Improvement in physician’s confidence after use of AI-QCT

**Figure 4:**
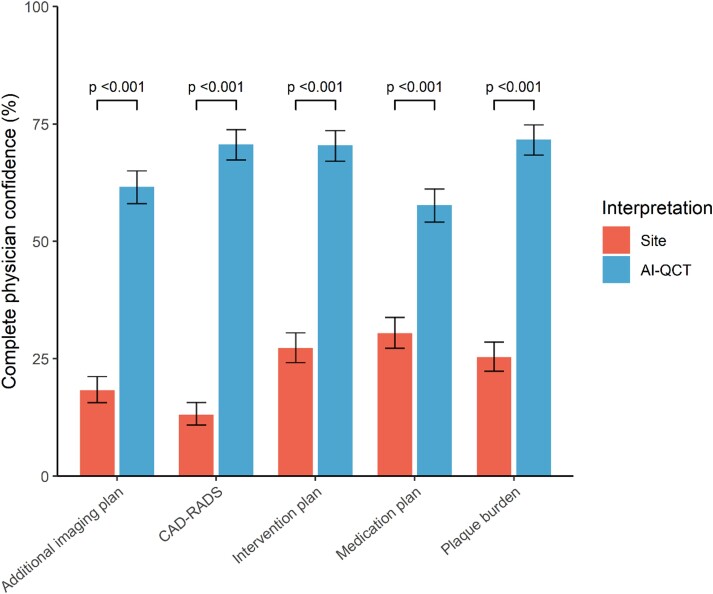
Physician’s confidence in care pathway with site CCTA assessment compared with AI-QCT assessment. Physician’s confidence in different steps of the care pathway with site interpretation and AI-QCT interpretation. AI-QCT, atherosclerosis imaging-quantitative computed tomography; CAD-RADS, Coronary Artery Disease-Reporting and Data System; CCTA, coronary CT angiography.

Compared with the site CCTA interpretation, AI-QCT analysis improved physician’s complete confidence two- to five-fold in every step of the care pathway, which was defined as a Likert confidence score of 5 out of 5 (*Figure 4*). Based on the conventional site assessment, there was complete physician confidence in the CAD-RADS score in 98 (13.1%) patients, which increased to 529 (70.5%) after AI-QCT interpretation (*P* < 0.001). For plaque burden, complete confidence increased from 190 (25.3%) patients with conventional site assessment to 521 (69.5%) with AI-QCT interpretation (*P* < 0.001). The number of patient assessments with complete physician confidence increased from 137 (18.3%) to 462 (61.6%), from 204 (27.2%) to 526 (70.1%), and from 228 (30.4%) to 432 (57.7%) for the additional imaging, intervention, and medication plans, respectively (*P* < 0.001 for all).

Overall, the assessment of AI-QCT was associated with at least one change in diagnosis (CAD-RADS or plaque burden), imaging, downstream testing, and medication in the majority (428; 57.1%; *P* < 0.001) of patients compared with the conventional site CCTA assessment (*Table 3*). When stratified according to the CAD-RADS score, the overall net change in the care pathway was higher in patients with obstructive CAD (159; 78.3%) than in patients with non-obstructive CAD (268; 49.2%; *P* < 0.001). A sensitivity analysis restricted to patients with suboptimal image quality showed generally similar results, but downstream changes in imaging were more prevalent in those with suboptimal image quality compared with those with optimal image quality (27.9 vs. 17.2%; *P* = 0.001; see Supplementary data online, *Table S1*).

## Discussion

In this real-world multi-centre study of 750 consecutive patients with suspected CAD, we observed that the use of AI-QCT, when compared with the conventional CCTA evaluation, resulted in major changes in physician confidence. Per assessment of the site physician, quantitative atherosclerosis assessment resulted in a reduction in patients indicated for additional non-invasive testing. Additionally, AI-QCT eliminated the expected need for coronary intervention in one in six patients, while medication prescription was increased in approximately one in four patients. Collectively, these data imply that AI-QCT may improve diagnostic certainty, may reduce non-invasive and invasive imaging, and may alter interventional procedural decision-making.

The current study findings are novel, as no prior study has investigated an integrated AI-enabled assessment of CAD simultaneously evaluating atherosclerosis, stenosis, and ischaemia. Our study results are additive to prior studies that have evaluated single-point computational technologies, such as FFR_CT_, on patient management. As an example, the FFR_CT_ RIPCORD study found that compared with CCTA site assessment alone, FFR_CT_ resulted in a change in management in 36% of patients.^19^ In contrast to the current study, the former study did not investigate the effect on downstream testing or specific medication prescription, given the inability of FFR_CT_ alone to quantify and characterize atherosclerosis or stenosis or CAD-RADS score. Data from the ADVANCE registry have further evaluated FFR_CT_.^20^ In a recent *post hoc* analysis from the CREDENCE and PACIFIC studies, AI-QCT_ISCHEMIA_ evaluation of coronary vessel-specific ischaemia has been shown to outperform FFR_CT_, when compared with an invasive FFR ≤0.8 reference standard.^13^ Beyond ischaemia, AI-QCT evaluation also includes stenosis and plaque assessment, which has robust prognostic value in a recent near-, intermediate-, and long-term follow-up study in 536 patients.^4^ Given the widespread adoption of FFR_CT_ in worldwide cardiology practice and the significantly lower rates of rejected studies by AI-QCT (5 vs. 19%),^13^ AI-QCT may allow a unique simultaneous approach to assess atherosclerosis, stenosis, and ischaemia. This study may provide an estimate of the impact of such an approach on clinical decision-making.

The observed reduction in need for downstream non-invasive testing in this study, predominantly driven by a reduction in myocardial perfusion imaging and FFR_CT_, may be a result of the included AI-QCT_ISCHEMIA_ evaluation and may have several benefits for patients and the healthcare system in general. CCTA now holds a Class I-A indication in the US Chest Pain guideline with the highest level of evidence. Given the rapid technological advancements in CCTA acquisition and analysis over the last years in conjunction with its strong prognostic relevance,^4,21^ present and future professional societal guidelines may allow for CCTA as paramount in CAD assessment.^1,2^ Thus, it would be of high value if CCTA can provide ischaemia assessment, given the non-negligible radiation dose and costs associated with SPECT, currently the most commonly used stress test in the USA.^22,23^ Considering the large and increasing part of patients suspected of CAD who will undergo CCTA imaging, the inclusion of routine ischaemia assessment in a more advanced CCTA analysis may reduce the need for SPECT, radiation, and costs.

We also observed that AI-QCT assessment reduced the need for coronary intervention. This practice, if enacted widely, might reduce unnecessary invasive procedures in patients with false-positive stress testing or for patients who would be referred by overestimation of coronary stenosis by human readers.^6,11,24,25^ Interestingly, AI-QCT not only reduced the physician decision to proceed with intervention by 16%, but also increased the proportion of patients with a predetermined type of intervention (PCI or CABG) from 27 to 54%. However, it should be noted that with the conventional CCTA approach, the number of patients requiring intervention might further decrease if additional functional or non-invasive tests were to yield normal results. It is likely that with the use of AI-QCT_ISCHEMIA_, physicians had more upfront certainty which lesions would require revascularization and thus had more clarity which type of coronary intervention to advise. In conjunction with the use of the SYNTAX score as advised by the SCCT expert consensus,^26^ AI-QCT may thus further improve pre-procedural planning of coronary revascularization compared with conventional CCTA assessment. Future studies evaluating AI-QCT_ISCHEMIA_, when combined with anatomic SYNTAX scores, now appear warranted.

The observed reclassification in CAD-RADS and plaque burden following AI-QCT analysis in the current study was largely driven by upwards reclassification between non-obstructive CAD-RADS categories and no and mild plaque burden, respectively. As illustrated in a recent PARADIGM *post hoc* analysis,^27^ the vast majority (87%) of the small plaques ≤50 ​mm^3^ identified by AI-QCT persist and, on average, may triple in size over a period of 4 years. Although a significant proportion of the patients with obstructive stenosis or a large plaque burden was also reclassified, the overall reclassification effect between AI-QCT and site CCTA interpretation was smaller than in patients with non-obstructive disease. The increased prescription of preventive medication, predominantly in patients with non-obstructive CAD, suggests that physicians and patients act upon these findings. Between the regular site CCTA assessment and the application of AI-QCT, the prescription rates of statins and aspirin increased by 28 and 23%, respectively. Results from prior studies such as SCOT-HEART have suggested that awareness of plaque burden by physicians and patients may both increase medication prescription as well as adherence and result in beneficial lifestyle changes.^28,29^ In addition to the notion that AI-QCT detects smaller plaque volumes than human readers,^10,27^ the comprehensive digital portal graphically outlining CCTA results may also underlie the further increased rate of medication prescription after AI-QCT assessment. The effect of the increased medication prescription on cardiovascular outcomes needs to be determined in randomized longitudinal studies.

This study has important implications for clinical practice and for healthcare payers: AI-QCT increased the number of treating physicians who had complete confidence in the diagnostic results by two- to five-fold. This increase in physician’s confidence may partly result from a comprehensive quantitative assessment of atherosclerosis, stenosis, and ischaemia with AI-QCT in contrast to solely visual stenosis estimations from conventional CCTA assessment. In addition to increasing physician’s confidence, systematic, graphically clear and simple reporting of CCTA results through AI-QCT may also improve patient health literacy, thereby encouraging adherence to medical therapy and salutary lifestyle habits. Further, implementation of routine AI-QCT analysis for CCTA can save a significant amount of time associated with manual grading of coronary stenosis and atherosclerosis, especially with the increasing number of plaque characteristics which can be derived from contrast-enhanced CCTA, such as low-density plaques, high-risk plaques, and others. Finally, AI-QCT can reduce inter-observer variability observed between different clinical readers, and reduce the overestimation of coronary stenosis by human readers.^4,6^

### Limitations

This study is not without limitations. Although this was a multi-centre study with 750 patients guided for care by five different centre’s physicians, individual physician behaviour may differ in other centres or in different countries outside the USA which may limit the generalizability of the findings. Furthermore, the clinical consequences of increased physicians’ confidence are unknown and the increase in physicians’ confidence observed in the current study might be different in other centres with less or more experience with the AI-QCT algorithm. The impact of AI-QCT was assessed using physician questionnaires, and the actual clinical management after downstream testing or invasive coronary angiography may have turned out different in some patients. CCTA acquisition was performed according to clinical guidelines; however, different CT scanners were used across the participating centres and there may have been differences in site-specific scan protocols despite adhering to the most recent SCCT guidelines, which may have affected study results.^16^ As AI-QCT has previously demonstrated high accuracy for assessment of stenosis and FFR for coronary ischaemia,^13,24,30^ the AI-QCT and conventional CCTA results were not compared with an invasive gold standard in this study. Finally, this study was not designed to assess the effect of routine use of AI-QCT on clinical and cardiovascular outcomes and the results should be considered hypothesis-generating. Randomized clinical studies are warranted to investigate whether AI-guided CCTA analysis and management will achieve more favourable outcomes than the current standard of care (NCT06112418).

### Conclusion

When compared with conventional CCTA interpretation by expert readers, AI-QCT enables a comprehensive assessment of atherosclerosis, stenosis, and ischaemia aligned with CAD-RADS that improves diagnostic certainty in a manner that may reduce the need for downstream non-invasive testing and might have consequences for therapeutic decision-making of medical and interventional therapies.

## Supplementary Material

jeae029_Supplementary_Data

## Data Availability

The data underlying this article will be shared on reasonable request to the corresponding author.
