# Supplementary material for: Impact of atherosclerosis imaging-quantitative computed tomography on diagnostic certainty, downstream testing, coronary revascularization, and medical therapy: the CERTAIN study
Source: Eur Heart J Cardiovasc Imaging. 2024 Jan 25;25(6):857–66. doi: 10.1093/ehjci/jeae029 (PMC11139521; doi:10.1093/ehjci/jeae029)
Supplement: jeae029_Supplementary_Data [file jeae029_supplementary_data.docx]

**ONLINE-ONLY SUPPLEMENTARY MATERIAL**

**Impact of atherosclerosis imaging-quantitative computed tomography on diagnostic certainty, downstream testing, coronary revascularization, and medical therapy: the CERTAIN study**

| **Content** | **Page** |
| --- | --- |
| Table S1. Change throughout the care pathway with AI-QCT compared to conventional site assessment, stratified by image quality | 2 |

**Table S1. Change throughout the care pathway with AI-QCT compared to conventional site assessment, stratified by image quality**

| Care pathway component | Overall  N = 750 | Suboptimal image quality  N = 430 | Optimal image quality  N = 320 | p-value |
| --- | --- | --- | --- | --- |
| Change in CAD-RADS score | 295 (39.3%) | 171 (39.8%) | 124 (38.8%) | 0.778 |
| Change in plaque burden interpretation | 197 (26.3%) | 118 (27.4%) | 79 (24.7%) | 0.397 |
| Change in imaging plan | 175 (23.3%) | 120 (27.9%) | 55 (17.2%) | 0.001 |
| Change in intervention plan | 127 (16.9%) | 83 (19.3%) | 44 (13.8%) | 0.045 |
| Change in medication prescription | 173 (23.1%) | 108 (25.1%) | 65 (20.3%) | 0.122 |
| Overall net change | **428 (57.1%)** | **260 (60.5%)** | **168 (52.5%)** | **0.029** |

Image quality was scored on a Likert scale and divided into suboptimal (Likert 1-3) and optimal image quality (Likert 4-5). AI-QCT, Atherosclerosis Imaging-Quantitative Computed Tomography.
